# Glycine increases fat‐free mass in malnourished haemodialysis patients: a randomized double‐blind crossover trial

**DOI:** 10.1002/jcsm.12780

**Published:** 2021-09-14

**Authors:** Laurence Genton, Daniel Teta, Menno Pruijm, Catherine Stoermann, Nicola Marangon, Julie Mareschal, Isabelle Bassi, Arelene Wurzner‐Ghajarzadeh, Vladimir Lazarevic, Luc Cynober, Patrice D. Cani, François R. Herrmann, Jacques Schrenzel

**Affiliations:** ^1^ Unit of Clinical Nutrition Geneva University Hospitals and University of Geneva Geneva Switzerland; ^2^ Service of Nephrology Cantonal Hospital of Sion Sion Switzerland; ^3^ Service of Nephrology University Hospital of Lausanne and University of Lausanne Lausanne Switzerland; ^4^ Service of Nephrology Geneva University Hospitals and University of Geneva Geneva Switzerland; ^5^ Service of Nephrology Geneva University Hospitals and Clinique of Champel Geneva Switzerland; ^6^ Genomic Research Lab and Service of Infectious Diseases Geneva University Hospitals and University of Geneva Geneva Switzerland; ^7^ EA 4466, Faculty of Pharmacy Paris University, and Clin Chem Lab, Cochin Hospital Paris France; ^8^ Louvain Drug Research Institute Metabolism and Nutrition Research Group, Walloon Excellence in Life Sciences and BIOtechnology (WELBIO) Université catholique de Louvain Brussels Belgium; ^9^ Department of Rehabilitation and Geriatrics Geneva University Hospitals and University of Geneva Geneva Switzerland

**Keywords:** Body composition, Amino acids, Lean body mass, Malnutrition

## Abstract

**Background:**

Protein energy wasting is associated with negative outcome in patients under chronic haemodialysis (HD). Branched‐chain amino acids (BCAAs) may increase the muscle mass. This *post hoc* analysis of a controlled double‐blind randomized crossover study assessed the impact of BCAAs on nutritional status, physical function, and quality of life.

**Methods:**

We included 36 chronic HD patient features of protein energy wasting as plasma albumin <38 g/L, and dietary intakes <30 kcal/kg/day and <1 g protein/kg/day. Patients received either oral BCAA (2 × 7 g/day) or glycine (2 × 7 g/day) for 4 months (Period 1), followed by a washout period of 1 month, and then received the opposite supplement (Period 2). The outcomes were lean body mass measured by dual‐energy X‐ray absorptiometry, fat‐free mass index measured by bioelectrical impedance, resting energy expenditure, dietary intake and appetite rating, physical activity and function, quality of life, and blood parameters. Analyses were performed by multiple mixed linear regressions including type of supplementation, months, period, sex, and age as fixed effects and subjects as random intercepts.

**Results:**

Twenty‐seven patients (61.2 ± 13.7 years, 41% women) were compliant to the supplementations (consumption >80% of packs) and completed the study. BCAA did not affect lean body mass index and body weight, but significantly decreased fat‐free mass index, as compared with glycine (coeff −0.27, 95% confidence interval −0.43 to −0.10, *P* = 0.002, respectively). BCAA and glycine intake had no effect on the other clinical parameters, blood chemistry tests, or plasma amino acids.

**Conclusions:**

Branched‐chain amino acid did not improve lean body mass as compared with glycine. Unexpectedly, glycine improved fat‐free mass index in HD patients, as compared with BCAA. Whether long‐term supplementation with glycine improves the clinical outcome remains to be demonstrated.

## Introduction

In patients undergoing chronic haemodialysis (HD), the prevalence of protein energy wasting (PEW) varies between 20% and 70%, depending on the nutritional marker considered.^1^ The aetiology of PEW is multifactorial and includes insufficient nutrient intake through anorexia, nutrient losses through dialysis, chronic inflammation due to co‐morbidities or HD *per se*, endocrine disorders (for instance, diabetes, insulin resistance, and hyperparathyroidism), metabolic acidosis, and anaemia.^2^, ^3^ Other elements like low circulating testosterone levels and advanced age may also contribute to PEW.^4^

Assessment of nutritional state in HD patients, and subsequent diagnosis of PEW, should be performed through a combination of laboratory, dietary, body composition, and functional measurements. The National Kidney Foundation^2^ and the International Society of Renal Nutrition and Metabolism (ISRNM)^5^ agree on the use of low albumin, low body weight, involuntary body weight loss, and low protein and energy intakes as common diagnostic criteria for PEW. Additional parameters are considered in one or the other definition, as, for instance, a decrease in muscle mass by the ISRNM and the recent update of the National Kidney Foundation.^6^

A decreased muscle mass is a hallmark of PEW, although not always assessed. It results from a decreased protein synthesis and myogenesis and an increased protein breakdown.^7^ A low muscle mass in HD patients has been associated with an increased risk of hospitalization and poor survival.^8^, ^9^ Nutritional treatments thus focus on improving body weight and muscle mass. Randomized double‐blind studies have shown that oral supplementation with a mixture of branched‐chain amino acids (BCAAs) can improve appetite, nutritional intake, plasma albumin, and lean body mass, as compared with basal values in patients undergoing chronic HD,^10^ with cancer^11^ or liver cirrhosis.^12^ One of these studies compared the impact of BCAA with an isocaloric placebo consisting of glycine, but only over 7 days,^11^ while the others used dextrose as placebo. Several meta‐analyses highlighted the anabolic potential of BCAA,^13^ and specifically leucine,^14^, ^15^ in elderly persons.

This *post hoc* analysis of a controlled double‐blind randomized crossover study assessed the impact of BCAAs on nutritional status, physical function, and quality of life in chronic HD patients. We hypothesized that BCAA supplementation over 4 months would improve nutritional status and eventually physical function and quality of life, as compared with glycine. This study allows fine‐tuning the type of amino acid required to improve nutritional state in HD patients.

## Study population and methods

This multicentric Swiss study (University Hospitals of Geneva, Clinic of Champel Geneva, University Hospital of Lausanne, Cantonal Hospital of Sion) took place from 1 August 2016 to 31 August 2019. The protocol was accepted by the local ethics committees (SNCTP000003307), and all included patients signed an informed consent. The trial was registered under clinicaltrials.gov, identifier: NCT 02962089.

### Study design

This study was a 9 month randomized, double‐blind crossover trial. HD patients with PEW received either oral BCAA or glycine for 4 months, followed by a washout period of 1 month, and then received the opposite supplement. The 4 month duration of each treatment relies on the study of Cano *et al*.^16^ It showed that the implementation of a nutritional support improved nutritional parameters, such as plasma albumin, with a plateau occurring at Month 4.

The primary outcome was gut microbiota composition, and the results are published elsewhere.^17^ This *post hoc* analysis evaluated the impact of these supplements on lean body mass measured by dual‐energy X‐ray absorptiometry, fat‐free mass index measured by bioelectrical impedance, resting energy expenditure, dietary intake and appetite rating, physical activity and function, quality of life, and blood parameters, which are detailed later.

Data and safety monitoring were performed throughout the study by an independent monitoring board. Adverse events were classified according to the Common Terminology Criteria for Adverse Events (CTCAE v5.0).^18^

### Study population

We included patients undergoing HD for ≥3 months, had not received systemic antibiotics in the previous month, and had features of malnutrition such as pre‐dialysis plasma albumin measured by bromocresol green <38 g/L without any known acute infection during the previous 2 weeks, and nutritional intakes <30 kcal/kg/day and <1 g protein/kg/day based on a 24 h dietary recall.^2^ The exclusion criteria were known cognitive or psychiatric disorder, poor compliance with drugs or HD, life expectancy <1 year, inadequate dialysis, low levels of plasma albumin due to other causes than nutrition, oral nutritional supplements or drugs with fibres or drugs affecting body composition since ≤1 month, known endocrine disorders altering energy metabolism, untreated or treated since ≤1 month, pregnancy, and breastfeeding.

### Study supplements

Supplements were produced as granules by the Pharmacy Bichsel (Interlaken, Switzerland) and conditioned in standardized 7 g amino acid packs (Ivers‐Lee AG, Burgdorf, Switzerland) according to the cGMP of the local regulatory authority. BCAA and glycine supplements were identical in taste and appearance. The patients were instructed to take the study supplements twice a day, about 30 min before breakfast and dinner. One pack of BCAA contained 3.62 g leucine, 1.94 g valine, and 1.45 g isoleucine, while one pack of glycine contained 7 g glycine. The cumulative daily dose of BCAA was in line with former randomized double‐blind placebo‐controlled studies including HD patients^10^ and patients with cancer,^11^ liver disease,^12^ or rheumatic disorders.^19^ Compliance to the study supplements was defined as over 80% of consumption over each 4 month period. It was systematically checked by counting the number of empty and full packs that the patient brought back every month.

Regarding the allocation of treatments, three lists of randomization were generated with random block sizes of 2 and 4, in order to obtain an equal proportion of each supplementation sequences for the University Hospitals of Geneva, the University Hospital of Lausanne, and the Cantonal Hospital of Sion. As the clinic of Champel joined as study centre in October 2017 to improve the recruitment, it could not benefit from a separate randomization list. We provided this centre with the two last supplementations of Lausanne and the four last supplementations of Sion.

### Outcome measurements

In addition to the parameters mentioned in the other paper,^17^ patients underwent measurements of body weight and composition, resting energy expenditure, dietary intake, physical activity and function, quality of life, and blood parameters at the start and end of each nutritional supplementation period, and for some parameters in between, as detailed in the Supporting Information, *Table* S1, but a least at the start and end of each supplementation. Adverse events were reported monthly.

### Body weight and composition

Body weight was measured at the start and end of each HD session. Inter‐dialytic weight gain was calculated as pre‐dialysis weight minus weight at the end of the previous dialysis. Body composition was assessed within 90 min of the end of the HD, by dual‐energy X‐ray absorptiometry (DXA) and bioelectrical impedance analysis (BIA).

One centre used a Lunar iDXA^®^ device (GE‐Lunar, Madison, WI, USA), and the other centres Hologic Discovery^®^ devices (Hologic, Waltham, MA, USA). A cross‐calibration between these devices, using a body composition phantom, showed that the Lunar iDXA underestimated lean soft tissue index by 1199.8 g, bone mineral content by 43.6 g, and overestimated fat mass by 420.4 g compared with the Hologic device. Thus, we corrected the Lunar measurements accordingly. Lean body mass, corresponding to the addition of lean soft tissue and bone mineral content, and fat mass were divided by height (m)^2^ and expressed as lean body mass and fat mass indices. This normalization was necessary to compare subjects with different heights.

Tetrapolar BIA was performed by Nutriguard^®^ (Data Input, Darmstadt, Germany). After cleaning the skin with 70% alcohol, adhesive electrodes were placed on the right hand and foot, or on the left if there was an arteriovenous fistula on the right arm, while the subject was lying on his back. If the subject had an arteriovenous fistula on the right arm, the measurements were the electrodes were placed on the left side. A generator applied an alternating electrical current of 50 kHz and 0.8 mA to these electrodes and measured resistance and reactance, which were used to calculate fat‐free mass by the Geneva formula.^20^, ^21^ Fat mass was obtained by subtracting fat‐free mass from body weight. Fat‐free and fat masses were expressed as indices, like the body composition measured by DXA.

### Venous blood parameters

We measured monthly pre‐dialysis haemoglobin, lymphocytes, transthyretin, C‐reactive protein, albumin (bromocresol green), urea, creatinine, and post‐dialysis urea, Kt∕V (K: dialyzer clearance of urea; t: dialysis time; and V: volume distribution of urea) as a marker of HD efficiency calculated with the Daugirdas formula^22^ (norm: single pool Kt∕V > 1.2) and normalized protein catabolic rate (nPCR). nPCR was calculated as follows: nPCR = 0.22 + (0.036 × intradialytic rise in blood urea nitrogen × 24)∕(intradialytic interval). At the start and the end of each 4 month treatment, we measured additionally total cholesterol, parathyroid hormone, and 25‐hydroxivitamin D, and serum bicarbonate as a marker of acid–base homeostasis. All these parameters were measured by routine methods. Plasma amino acids were measured by ion exchange chromatography with post‐column derivatization using ninhydrin.

### Nutritional intakes, appetite, and resting energy expenditure

At the start of each treatment and every 2 months, actual calorie and protein intakes were calculated by a 3 day food diary (two weekdays, including 1 day of HD, and 1 day of the weekend). The dietician checked with the patient the adequacy of intake reporting and analysed the data. Resting energy expenditure was measured at rest, after an overnight fast, by indirect calorimetry (Quark RMR^®^, Cosmed, Pavone, Italy).

### Physical function and activity

Handgrip strength was assessed with the Baseline^®^ hydraulic hand dynamometer (12‐0240, White Plains, NY, USA), in the second handle position. Measurements were performed in the sitting position, with the forearm and wrist resting on a table in neutral position and the elbow flexed at about 90°, three times with each hand.^23^ The maximum value obtained from both hands was considered for analysis as described elsewhere.^24^ Physical activity was assessed by a pedometer (Yamax Digiwalker SW‐200^®^, London, UK). This device was worn at the waistband for 7 days. The Yamax pedometers were shown to be among the best pedometers regarding accuracy and reliability of step counting compared with hand counter.^25^, ^26^

### Quality of life

Quality of life was assessed by the RAND 36‐Item Short Form Health Survey, available publicly on Internet.^27^ It evaluates eight health domains with each domain scoring from 0 (very unfavourable) to 100% (very favourable) of the total possible score: physical functioning, limitations due to physical health, limitations due to emotional health, energy/fatigue, emotional well‐being, social functioning, pain, general health, and health change.

### Statistics

Results were expressed as mean (standard deviation) or *n* (frequency). BCAA–glycine corresponds to the group who started the study with the BCAA supplement and glycine–BCAA to the group who started with the glycine supplement. Normality of distribution of continuous parameters was checked by Shapiro–Wilk tests. Continuous parameters at baseline were compared between both groups with unpaired *t*‐tests or Wilcoxon rank‐sum tests, as appropriate, and ordinal parameters with Fisher's exact tests. Significance was set at *P* < 0.05 and corrected for multiple comparisons by the Benjamini–Hochberg method.^28^

**Figure 1:**
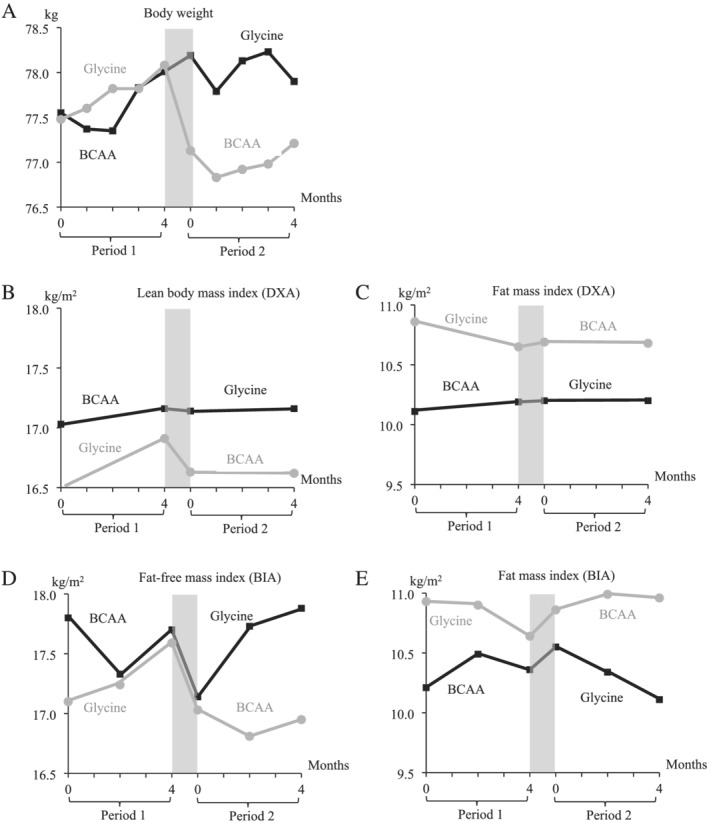
Line plot showing the evolution of *(A)* weight (kg), *(B)* lean body mass index (kg/m^2^), *(C)* fat mass index (DXA), *(D)* fat‐free mass index (kg/m^2^/day), and *(E)* fat mass index (BIA) (kg) in the BCAA–glycine group (black squares and line) and in the glycine–BCAA group (grey dots and line). The grey zone indicates the washout period, which occurred for each patient between Months 4 and 5.

The impact of the type of supplements (BCAA or glycine) on each continuous parameter was analysed by multiple mixed linear regression models, as suggested by Dwan *et al*.^29^ and others.^30^, ^31^, ^32^, ^33^ These analyses take into account the crossover design of the study and include all values assessed at all time points. Analyses were performed according to the latest CONSORT statement for randomized crossover trials,^29^ which recommends to include only the patients who have completed the trial, thus to perform a per‐protocol analysis, and not to test for the carry‐over effect. Besides the type of supplements, the models included the period (first 4 months of supplementation vs. the following 4 months, cf. *Figure* 1), the months (0–4 in each period), the age and the sex as fixed effects, and subjects as random intercepts. Outcomes that were not normally distributed were normalized using the Stata's ‘ladder’ command, in order to be used in the mixed regression models. In case of significance of a model, we added a ‘supplementation × months’ interaction to evaluate at what months the differences occurred. The significance of mixed regression models was set at *P* < 0.05 and also corrected by the Benjamini–Hochberg method.^28^ For the outcome body composition, whether measured by DXA or BIA, we repeated the models with adjustment for baseline values. We also performed the models with a ‘period × months’ interaction, in order to evaluate whether the changes of the measured parameters depended on the period of supplementation.

In case of a ‘month’ effect with a *P* value <0.01 in any of the multiple regression models significant after correction by the Benjamini–Hochberg method, we repeated the analyses separately by type of supplementation. This was performed in order to see if the BCAA or the glycine increased or decreased a given parameter, compared with baseline.

Calculation of sample size was performed for the primary outcome, that is, gut microbiota composition, as described elsewhere^17^ and resulted in a need of 36 patients per group. We had also performed a sample size calculation for lean body mass, based on a previous crossover study performed in HD patients by Hiroshige *et al*.^10^ As the authors did not report the differences of lean body mass between the BCAA and placebo groups at the end of the study, we used the difference between the malnourished HD patients under placebo and the well‐nourished HD patients and, as standard deviation for lean body mass, the highest value of all groups at baseline. Thus, we anticipated that lean body mass would increase by 5.0 ± 6.5 kg over 4 months with the BCAA treatment, which corresponds to the rounded values of Hiroshige *et al*. at baseline. We considered a dropout rate of 30% (15% for death, 10% for non‐compliance, and 5% for kidney transplantation). The final number of subjects needed was calculated using the following published formula^34^: *N* final = *N* power∕((1—death risk)^*i*^ * (1—non‐compliance)^*i*^ * (1—transplantation)^*i*^), where *i* is equal to duration of the study, rounded up to 1 year in this study. Thus, we obtained a needed total sample size of 19 subjects for this secondary endpoint.

## Results

We screened 303 patients, included and randomized 37 patients, but only 36 patients (13 women and 23 men) started the study and had baseline values.^17^ This was because one patient signed the informed consent but withdrew his consent before having performed the baseline tests. Thus, his treatment was attributed to another patient. The adverse events that occurred during the study in the 36 patients are shown in *Table* 1 and were not statistically significant between both supplementations. All the adverse events reported as serious were those prompting for the hospitalization of the patient. No death occurred during the study.

**Table 1:** Adverse events during the study supplementation (*n* = 36)

System organ class	CTCAE term	BCAA	Glycine	Washout
Serious adverse events				
Gastrointestinal disorders	Colonic haemorrhage	1	1	
	Gastritis	1		
Cardiac disorders	Unstable angina pectoris	1	1	
Vascular disorders	New fistula or dialysis catheter	1	1	
Respiratory, thoracic, and mediastinal disorders	Pneumonia		1	
	Acute pulmonary oedema	1		
Nervous system disorders	Dyskinesia		1	
Musculoskeletal disorders	Fall with prepatellar haematoma			1
Other adverse events				
Gastrointestinal disorders	Dyspepsia		2	
	Nausea or vomiting	1	6	3
	Abdominal pain or bloating	1	2	
	Diarrhoea	4	2	2
	Constipation		1	
	Rectal bleeding			1
	Ileocaecal obstruction			1
Renal and urinary disorders	Supplemental dialysis sessions^a^	10	11	1
	Urinary tract infection	1		1
Cardiac disorder	Bradycardia during HD	1		
	Tachycardia before HD	1		
	Angina pectoris		1	1
Vascular disorders	Hypertension during HD	3	2	
	Hypotension during HD	13	11	
	Change of dialysis catheter	2		
	Vascular stenosis	3	2	1
Respiratory, thoracic, and mediastinal disorders	Acute pulmonary oedema		1	
	Upper airway infection	4	5	4
	Influenza	1	1	
	Pneumonia	1		1
	Sore throat	1		
	Epistaxis	2	1	
Nervous system disorders	Syncope during HD	1	1	
	Syncope after HD		1	
	Vertigo	1		
Eye disorders	Vitreous haemorrhage	1		
	Conjunctival haemorrhage	1		
	Scleral disorder			1
Injury, poisoning, and procedural complications	Fall at home	1		3
Musculoskeletal and connective tissue disorders	Arthralgia	3	1	
	Back pain		1	1
	Chest wall pain	1		
	Cramp/pain in legs during HD	2	4	
	Restless leg syndrome	1	1	1
	Carpal tunnel syndrome		1	
	Diabetic foot		1	
	Plantar fasciitis	1		
	Fractures of lower extremity	3		
Skin and subcutaneous tissue disorders	Pruritus		1	
Psychiatric disorders	Agitation		1	
	Insomnia			
Metabolism and nutrition disorders	Anorexia		1	
General disorders	Fatigue	5	1	
Surgical procedures	Jaw cyst removal		1	
	Elective aortic replacement		1	
	Cryotherapy for actinic keratosis	1		
	Sclerotherapy for lymphangioma	1		
	Endoscopic resection of bladder	1		
Plasma abnormalities	Hypercalcaemia		1	
	Hyperkalaemia	1	1	1
	Hyperphosphataemia or hypophosphatemia	2		1
	Hyperglycaemia or hypoglycaemia	1	1	
	High or low parathormone	1	1	
	Disturbance of liver tests			1

BCAA, branched‐chain amino acid; CTCAE, Common Terminology Criteria for Adverse Events; HD, haemodialysis.

The International Conference on Harmonization Good Clinical Practice defines the following terms: ‘An adverse event (AE) is any unfavourable and unintended sign (including an abnormal laboratory finding, for instance), symptom, or disease (including the worsening of the existing disease) occurring in a patient during the study period, whether or not considered related to the study drug. A serious adverse event (SAE) is defined as any of the untoward medical incident that results in death, is life threatening, and requires inpatient hospitalization or prolongation of existing hospitalization (life threatening is defined as any adverse experience that places the patient, in the view of the investigator, at immediate risk of death)’. Adverse events are expressed as number of episodes.

^a^ Supplemental dialysis sessions due to fluid overload, which occurred in four patients: one patient required eight supplemental sessions under BCAA, eight under glycine, and one in the washout period; two patients had one supplemental dialysis session under BCAA and one under glycine each; and finally, one patient had one supplementary dialysis session under glycine.

Of the 37 patients randomized initially, 27 (11 women and 16 men) finished the study and were analysable, that is, 15 patients in the BCAA–glycine group (4 women and 11 men) and 12 patients in the glycine–BCAA group (7 women and 5 men). Their demographic characteristics and baseline health parameters are shown in *Tables* 2 and 3, respectively. Baseline muscle mass was low, that is, <17 kg/m^2^ in four men and <15 kg/m^2^ (Cederholm *et al*.^35^) in four women, when relying on Swiss reference values of fat‐free mass index.^36^ The washout period (time from the last treatment taken of Period 1 until the first treatment of Period 2) lasted 31 ± 4 days. The plasma amino acid profile at baseline did not differ between the groups (*Table* S2).

**Table 2:** Demographic characteristics of the patients (*n* = 27)

	Total	BCAA–glycine	Glycine–BCAA	*P* ^a^
	(*n* = 27)	(*n* = 15)	(*n* = 12)	
Mean (SD) age (years)	61.2 (13.7)	63.3 (13.4)	58.6 (14.2)	0.390
Mean (SD) duration of haemodialysis (months)	25.4 (21.1)	29.3 (21.1)	20.7 (21.0)	0.302
Gender				0.130
Female	11 (41)	4 (27)	7 (58)	
Male	16 (59)	11 (73)	5 (42)	
Site				0.870
Geneva^b^	15 (56)	9 (60)	6 (50)	
Lausanne	5 (19)	3 (20)	2 (17)	
Sion	7 (25)	3 (20)	4 (33)	
Aetiology of kidney failure^c^				0.525
Diabetes (Types 1 and 2)	9 (25)	6 (29)	3 (19)	
Hypertension	17 (47)	7 (33)	10 (63)	
Polycystic kidney disease	4 (11)	3 (14)	1 (6)	
Chronic glomerulonephritis	4 (11)	4 (19)	1 (6)	
Other	2 (6)	1 (5)	1 (6)	
Type of dialysis				0.487
Haemodiafiltration	25 (93)	13 (80)	12 (100)	
Haemofiltration	2 (7)	2 (20)	0	
Access				0.797
Native arteriovenous fistula	21 (78)	12 (80)	9 (75)	
Prothetic arteriovenous fistula	1 (4)	0	1 (8)	
Central catheter	5 (18)	3 (20)	2 (17)	
Prescribed dialysis time/session				0.487
4 h, 3× per week	25 (93)	13 (80)	12 (100)	
3.5 h, 3× per week	2 (7)	2 (20)	0	
Dialysis membrane				
Copolymer of acrylonitrile and methylsulfonate	4 (5)	2 (20)	2 (17)	0.611
Polysulfone or polyamide	23 (85)	13 (80)	10 (83)	

BCAA, branched‐chain amino acid; SD, standard deviation.

Data are expressed as number (%), unless stated otherwise.

^a^ Comparisons between groups by Fisher's exact test or unpaired *t*‐tests as appropriate. With the Benjamini–Hochberg method, significance was corrected to *P* < 0.005.

^b^ Patients included in the clinic of Champel (*n* = 6) performed all their study assessments at the University Hospitals of Geneva and were dialyzed by a fellow of the University Hospital of Geneva (N.M.). They were thus included in the site ‘Geneva’.

^c^ One patient can have several aetiologies for his kidney failure, explaining that the *n* may be higher than 27, 15, or 12 for the total patient, patients in the BCAA–glycine group, and patients in the glycine–BCAA group, respectively.

**Table 3:** Baseline health parameters (*n* = 27)

	BCAA–glycine (*n* = 15)	Glycine–BCAA (*n* = 12)	*P* ^a^
	Mean ± SD	Mean ± SD	
Anthropometry and body composition			
Height (cm)	167. ± 9.5	166.0 ± 8.9	0.703
Body weight (kg)	77.6 ± 15.6	77.5 ± 14.2	0.991
Body mass index (kg/m^2^)	27.7 ± 5.1	28.0 ± 4.1	0.854
Body composition			
DXA lean soft tissue mass (kg)	47.9 ± 8.9	45.9 ± 10.5	0.595
DXA fat mass (kg)	28.0 ± 9.4	29.6 ± 7.2	0.623
DXA bone mineral content (kg)	2.1 ± 0.6	2.1 ± 0.6	0.879
DXA lean body mass index (kg/m^2^)^b^	17.0 ± 2.3	16.5 ± 2.5	0.559
DXA fat mass index (kg/m^2^)	10.1 ± 3.6	10.9 ± 2.9	0.565
BIA fat‐free mass (kg)	49.6 ± 9.7^c^	47.6 ± 10.1	0.613
BIA fat mass (kg)	27.9 ± 10.1^c^	29.9 ± 8.1	0.593
BIA fat‐free mass index (kg/m^2^)	17.8 ± 2.4^c^	17.1 ± 2.3	0.458
BIA fat mass index (kg/m^2^)	10.2 ± 3.9^c^	10.9 ± 3.1	0.612
Blood parameters			
Haemoglobin (g/L)	109.5 ± 20.3	103.1 ± 14.9	0.367
Pre‐dialysis urea (mmol/L)	18.5 ± 6.6	19.2 ± 5.8	0.795
Creatinin (μmol/L)	678.6 ± 216.9	687.1 ± 242.0	0.924
nPCR	1.0 ± 0.2	1.1 ± 0.3	0.297
Kt∕V_urea_	1.6 ± 0.3	1.8 ± 0.4	0.295
Bicarbonate (mmol/L)	22.2 ± 3.4	24.2 ± 2.0	0.080
Albumin (g/L)	38.3 ± 3.2	39.1 ± 2.7	0.484
Transthyretin (mg/L)	295.6 ± 60.8	340.3 ± 60.5	0.069
C‐reactive protein (g/L)	15.5 ± 21.3	5.9 ± 7.6	0.169
Cholesterol (mmol/L)	4.4 ± 0.7	4.2 ± 1.1	0.418
Parathyroid hormone (pmol/L)	33.3 ± 31.8	44.5 ± 69.7	0.582
25‐OH vitamin D (nmol/L)	75.5 ± 30.6	76.2 ± 34.2	0.956
Intake and appetite			
Kilocalories (kcal/kg)	22.1 ± 8.0	21.9 ± 5.1	0.924
Protein (g/kg)	0.9 ± 0.3	0.9 ± 0.3	0.966
Appetite rating (mm)	60.6 ± 15.3	55.4 ± 9.8	0.315
Indirect calorimetry			
VCO_2_ (mL/min)	164.7 ± 37.7	181.9 ± 39.3	0.257
VO_2_ (mL/min)	186.5 ± 51.7	210.8 ± 58.1	0.263
Respiratory quotient	0.9 ± 0.3	0.9 ± 0.1	0.571
Resting energy expenditure (kcal/day)	1309.1 ± 338	1472.3 ± 384.8	0.252
Physical function			
Handgrip strength (kg)	25.1 ± 9.7	23.3 ± 12	0.681
Pedometry (steps/day)	3143.7 ± 2983.4	4189.1 ± 3136.3	0.385
Quality of life			
General health (0–100%)	53.3 ± 19.8	49.6 ± 19.5	0.627
Health change (0–100%)	55.0 ± 30.2	66.7 ± 24.6	0.290

BCAA, branched‐chain amino acid; BIA, bioelectrical impedance analysis; DXA, dual‐energy X‐ray absorptiometry; nPCR, normalized protein catabolic rate; SD, standard deviation.

^a^ Comparisons between groups by *t*‐tests. With the Benjamini–Hochberg method, significance was corrected to *P* < 0.001.

^b^ Lean body mass = lean soft tissue + bone mineral.

^c^ *N* = 13.

### Impact of branched‐chain amino acid vs. glycine supplementation

*Figure* 1 shows the evolution of body weight and body composition during the study. The individual patient line plots are shown in *Figure* S1. The differences of the outcomes between Months 4 and 0 by supplementation are shown in *Table* S2. Mean inter‐dialytic weight gain for the BCAA and glycine supplementation was +1.79 and +1.83 kg during Period 1 and +1.66 and +1.95 kg during Period 2, respectively. It was not different between both supplementations in the multiple mixed linear regression model, after correction for multiple analyses (*P* = 0.025).

The multiple mixed linear regression models showed that fat‐free mass index measured by BIA, but not the lean body mass index measured by DXA nor the body weight (*Table* 4), decreased with the BCAA in comparison with glycine. These differences remained significant after correction for multiple analyses. The inclusion of a ‘supplementation × months’ interaction was significant only for the fat‐free mass index measured by BIA. It highlighted that under BCAA, fat‐free mass decreased between Months 0 and 2 [coeff −0.46, 95% confidence interval (CI) −0.86 to −0.06, *P* = 0.023] and between Months 0 and 4 (coeff −0.44, 95% CI −0.84 to −0.04, *P* = 0.029), as compared with the glycine. Neither the BCAA nor the glycine had a significant impact on fat mass index, whether measured by DXA or BIA (*Table* S3). When adjusting the mixed linear regression models for the baseline body composition measured by BIA, BCAA supplementation still decreased fat‐free mass index (coeff −0.30, 95% CI −0.47 to −0.14, *P* < 0.001) and additionally increased fat mass index as compared with the glycine (coeff 0.32, 95% CI 0.14 to 0.50, *P* = 0.001).

**Table 4:** Multiple mixed linear regressions including period, supplementation, months, age, and sex as fixed effects and subjects as random intercepts to predict body weight, lean body mass index, and fat‐free mass index

		Coefficient	95% CI	*P*	*P* model
Body weight (kg)^a^					0.052
Supplementation	Glycine	0			
	BCAA	−0.59	(−0.95 to −0.22)	0.002	
Months	0	0			
	1	−0.20	(−0.78 to 0.37)	0.489	
	2	−0.04	(−0.61 to 0.54)	0.896	
	3	0.14	(−0.44 to 0.72)	0.633	
	4	0.21	(−0.37 to 0.80)	0.476	
Period	1	0			
	2	−0.15	(−0.54 to 0.25)	0.469	
Age		−0.05	(−0.44 to 0.35)	0.816	
Sex	Female	0			
	Male	9.41	(−1.53 to 20.35)	0.092	
Lean body mass index by DXA (kg/m^2^)^a^					0.280
Supplementation	Glycine	0			
	BCAA	−0.07	(−0.27 to 0.13)	0.520	
Months	0	0			
	4	0.14	(−0.06 to 0.34)	0.165	
Period	1	0			
	2	0.01	(−0.20 to 0.21)	0.967	
Age		−0.03	(−0.09 to 0.04)	0.420	
Sex	Female	0			
	Male	1.68	(0.02 to 3.34)	0.047	
Fat‐free mass index by BIA (kg/m^2^)^b^					<0.001
Supplementation	Glycine	0			
	BCAA	−0.27	(−0.43 to −0.10)	0.002	
Months	0	0			
	2	−0.08	(−0.28 to 0.13)	0.469	
	4	0.18	(−0.02 to 0.38)	0.084	
Period	1	0			
	2	−0.11	(−0.28 to 0.05)	0.183	
Age		−0.01	(−0.06 to 0.05)	0.847	
Sex	Female	0			
	Male	2.36	(0.95 to 3.78)	0.001	

BIA, bioelectrical impedance analysis; BCAA, branched‐chain amino acid; CI, confidence interval; DXA, dual‐energy X‐ray absorptiometry.

Period 1: first 4 months of the study; Period 2: second 4 months of the study, after the washout. With the Benjamini–Hochberg method including analysis of these outcomes and those presented on *Table* S3, significance for supplementation was corrected to <0.005, leaving a significant negative impact of BCAA supplementation on body weight and fat‐free mass index by BIA, as compared with the glycine.

^a^ Weight and DXA: 27 patients and 108 measurements.

^b^ BIA: 26 patients and 155 measurements.

Blood parameters, intake, appetite, resting energy expenditure, physical function, and quality of life did not differ between the supplementations (*Table* S3). The mean calorie and protein intake stayed between 21–23 kcal/kg/day and 0.80–0.82, respectively, throughout the study and whatever the supplementation was. Regarding the plasma amino acid profile (*Table* S4), the BCAA supplementation decreased the plasma concentration of aspartate but increased that of valine. However, these differences disappeared when the *P* value was corrected for multiple testing.

The inclusion of a ‘period × months’ interaction was not significant in any of the previously mentioned models. We thus decided to focus on the analyses without interactions.

### Impact of branched‐chain amino acid or glycine supplementation vs. baseline values

The ‘month’ effect was <0.01 for fat‐free mass index, pre‐dialysis urea, nPCR, and the handgrip strength. A line plot shows the evolution of these parameters (*Figure* S2). Thus, we repeated the multiple mixed linear regression models separately by type of supplementation for these parameters.

As compared with baseline values, BCAA decreased the fat‐free mass index on Month 2 (coeff −0.31, 95% CI −0.54 to −0.79, *P* = 0.009). BCAA increased the normalized handgrip strength on Month 3 (coeff 0.15, 95% CI 0.01 to 0.29, *P* = 0.046), and, already by Month 1, the pre‐dialysis urea (2.19, 95% CI 0.05 to 4.33, *P* = 0.045) and the nPCR (2.19, 95% CI 0.05 to 4.33, *P* = 0.045 and coeff 0.15, 95% CI 0.04 to 0.26, *P* = 0.006, respectively).

Compared with Month 0, glycine increased the fat‐free mass index on Month 4 (coeff 0.40, 95% CI 0.10 to 0.69, *P* = 0.009) and increased, by Month 1 already, the handgrip strength (coeff 0.11, 95% CI 0.01 to 0.22, *P* = 0.048), the pre‐dialysis urea (4.33, 95% CI 2.41 to 6.24, *P* < 0.001), and the nPCR (0.20, 95% CI 0.10 to 0.294, *P* < 0.001).

## Discussion

This randomized, double‐blind crossover trial in patients under chronic HD surprisingly showed a decrease in fat‐free mass index with a 4 month supplementation of BCAA, in contrast to glycine, in HD patients.

To our knowledge, only three randomized controlled double‐blind trials evaluated the impact of BCAA on nutritional status in patients. The BCAA dose was always around 14 g/day, which is similar to our study. Hiroshige *et al*. showed in Japanese HD patients that BCAA over 6 months improved anorexia, calorie and protein intake, and BIA‐measured fat mass and fat‐free mass, as compared with basal values.^10^ In oncological anorexic patients, BCAA over 7 days, but not the placebo containing only glycine, increased plasma concentration of large neutral amino acids, calorie intake, and appetite.^11^ In patients with liver cirrhosis, BCAA over 1 year increased fat mass, plasma albumin, appetite, and physical function, but nutritional intake was not assessed.^12^ These results contrast with our study, where BCAA had no impact on body composition nor albumin and where BCAA decreased calorie intake. However, the two latter studies^11^, ^12^ were performed in a parallel design and in patients suffering other diseases with potentially different metabolic responses to supplementations than HD patients.

The study of Hiroshige *et al*. was performed in a crossover design,^10^ as ours, but included no washout period and dextrose was used as placebo. Compared with their study, our HD patients had a higher body mass index and plasma albumin at baseline but a lower calorie intake (∼22 vs. 26 kcal/kg body weight). We hypothesize that our patients were in a better nutritional status, with less room for improvement with BCAA. Their low calorie intake may be related to their low resting energy expenditure (<20 kcal/kg/day) and low physical activity. These elements were not reported in the study of Hiroshige *et al*.^10^ In their study, nutritional parameters improved with BCAA as compared with baseline but were not statistically different between the groups treated with BCAA vs. dextrose, which questions the superiority of BCAA. Thus, due to differences in patient population, type of nutritional supplementation, and statistical method (ANOVA vs. multiple mixed linear regression), our results cannot be compared with theirs.

In our study, all amino acid levels were in the normal range at baseline except citrullinaemia in both groups. This is because the kidney is the main organ in citrulline removal.^37^ Glycine supplementation did not increase plasma glycine concentration nor did BCAA supplementation increase plasma valine, leucine, and isoleucine concentrations. This may be due to the time between the administration of supplements and blood sampling. It has been shown that amino acid levels return to basal values within 3–4 h after oral bolus administration^38^ due to their rapid use by peripheral tissues, as, for instance, the gut, the liver, or the muscle.

The benefit of glycine on fat‐free mass was unexpected. Glycine is a non‐essential amino acid and is a precursor or component of molecules such as glutathione, haem, collagen, creatine, and purines.^39^ Animal studies, published after this study, had been launched and have shown that glycine can counteract muscle wasting in models of sepsis, energy restriction, and cancer cachexia.^40^, ^41^, ^42^, ^43^ Low plasma glycine concentrations have also been associated with increased insulin resistance,^44^, ^45^ an aetiological factor of PEW. The mechanisms leading to increased lean components are unclear, but oral glycine could affect fat‐free mass directly and/or through changes at the gut barrier level.^46^, ^47^ In a recent review, Koopman *et al*. suggested that glycine decreases cell damage, oxidative stress, and the production of pro‐inflammatory cytokines; overcomes the anabolic resistance to leucine in wasting models; and increases the protein synthesis in many cell types including skeletal muscle cells.^42^ Glycine has also a cytoprotective effect on intestinal epithelial cells by reducing oxidative damage,^48^ which could decrease gut permeability and thus prevent systemic inflammation, a driver of PEW.^49^ The bioavailability of dietary glycine for the intestinal epithelium depends on the gut microbiota, which can use glycine for the growth of specific strains or the production of metabolites.^50^ In summary, the benefit of glycine on fat‐free mass in HD patients may occur through anti‐inflammatory and cytoprotective properties, as well as decreased insulin resistance.

The fact that glycine had no impact on DXA‐measured lean body mass, in contrast to BIA‐measured fat‐free mass, could be explained by fewer DXA than BIA measurements, which increases the variance. The component of fat‐free mass affected by the glycine supplementation is unclear. Fat‐free mass consists mostly of water and protein, and to a minor extent glycogen, mineral, and essential lipids. We have considered the possibility that glycine increases the total body water and not the protein mass *per se*. However, glycine, as compared with BCAA supplementation, resulted in a similar inter‐dialytic weight gain, a similar number of supplemental dialysis sessions, and no increased occurrence of acute pulmonary oedema.

The high doses of amino acids received by our patients corresponded to the doses given in previous randomized controlled trials. No death occurred during the study. Eleven patients required hospitalization for serious adverse events, but none were related to the supplementation. Six patients under glycine experienced nausea or vomiting, which opens the question of a potential glycine‐related side effect. Indeed, the use of glycine as a distension solution in surgery has been associated with hyponatraemia and subsequent brain oedema.^51^ However, none of our patients developed hyponatraemia during glycine supplementation. Furthermore, three of the six patients also complained about nausea and vomiting during the washout period. It is therefore difficult attribute these gastrointestinal symptoms to glycine. Nausea is a frequent complaint of HD patients and may be related to the chronic uraemic state. There was no increased use of oral anti‐diabetic drugs or insulin dosage with any treatment, although this could be expected in view of the BCAA‐mediated increase in insulin resistance described in animal models.^52^, ^53^ The component of fat‐free mass affected by the glycine supplementation is unclear. Fat‐free mass consists mostly of water and proteins, and to a minor extent glycogen, minerals, and essential lipids. We have considered the possibility that glycine increases the total body water and not the protein mass *per se*. However, when considering only the 27 patients of the per‐protocol analysis, glycine, as compared with BCAA supplementation, resulted in a similar inter‐dialytic weight gain, a similar number of supplemental dialysis sessions (9 sessions under BCAA and 10 under glycine), and no increased occurrence of acute pulmonary oedema.

### Strengths and limitations

The strength of this study lies in its randomized, double‐blind crossover design. The included patients were compliant with their treatments as confirmed by the counting of the empty packs and by the increase in pre‐dialysis urea, the waste product of nitrogen, and the nPCR with both supplements. There are several limitations in this study. First, we had no control group, that is, a group who did not receive any amino acid supplement. This is because we had hypothesized that glycine was an adequate placebo, with no effect on lean body mass. Second, due to the crossover design, we could compare the differential impact of the supplementations on the nutritional state and clinical outcomes during the study, but we cannot evaluate their impact on the longer term, as all patients have received both treatments for 4 months. Third, the protein intake could have been too low to stimulate optimally muscle anabolism. It is in line with the US Recommended Dietary Allowance^54^ but below the recommendations for HD patients.^1^, ^55^ Finally, the sample size was small but in line with our power computation.

## Conclusion

Glycine, but not BCAA, supplementation over 4 months, improves fat‐free mass measured by BIA in HD patients. These findings confirm recent animal studies showing the potential of glycine to counteract the anabolic resistance to leucine in case of wasting. Whether glycine in the long term improves the muscle and function in other patients with chronic diseases and the subsequent clinical outcome remains to be demonstrated.

## Conflict of interest

L.G. has received grants from the Swiss National Science Foundation, Alfred and Alice Lachmann Foundation, and Fresenius Kabi; speaker honoraria from Fresenius Kabi; advisory honoraria from Baxter and Abbott; and travel grants from Nestlé Health Science and Abbott. P.D.C. is a co‐founder of A‐Mansia Biotech S.A. (Belgium) and owner of several patents concerning the use of specific bacteria or components on the treatment of obesity, diabetes, and cardiometabolic disorders. D.T. has received grants from Fresenius Medical Care, Amgen, and Baxter; speaker honoraria from Fresenius Medical Care, B. Braun, Abbott International, Baxter, Genzyme, and Sanofi Aventis; travel grants for Fresenius Medical Care, Amgen, and Vifor; and a grant for teaching material from Abbott International. M.P., C.S., N.M., J.M., I.B., A.W.‐G., V.L., L.C., F.H., and J.S. have no conflicts of interest.

## Funding

This study was financially supported by the Swiss National Science Foundation (Schweizerischer Nationalfonds zur Förderung der Wissenschaftlichen Forschung; 320030_163144), Alfred and Alice Lachmann Foundation, and Fresenius Kabi Deutschland GmbH. P.D.C. is senior researcher from the Fonds De La Recherche Scientifique ‐ FNRS (FNRS FRFS‐WELBIO) and recipient of the grant WELBIO‐CR‐2019C‐02R. The funding source had no role in the design and conduct of the study, analysis or interpretation of the data, or preparation of final approval of the manuscript prior to publication.

## Supporting information


**Table S1.** Study schedule detailing the frequency of the different assessments.
**Table S2.** Differences of the outcomes between month 4 and 0, by supplementation.
**Table S3.** Baseline plasma amino acid profile.
**Table S4.** Multiple mixed linear regressions including period (first 4 months vs. following 4 months), supplementation (branched chain amino acids vs. glycine) and months as fixed effects, and subjects as random intercepts, to predict outcomes other than lean body mass index and fat‐free mass index (*n* = 27).
**Table S5.** Multiple mixed linear regressions including period (first 4 months vs. following 4 months), supplementation (branched chain amino acids vs. glycine) and months as fixed effects, and subjects as random intercepts, to predict plasma amino acid concentrations (*n* = 26).
**Figure S1.** Individual patient lines plots for body weight (A), lean body mass index (DXA) (B), fat mass index (DXA) (C), fat‐free mass index (BIA) (D) and fat‐mass index (BIA) (E). The gray line is used for the BCAA supplementation and the black line for the glycine supplementation. Plain lines correspond to the BCAA‐glycine group, while the dashed lines correspond to the Glycine‐BCAA group.
**Figure S2.** Line plot showing the evolution of handgrip strength (squared root (handgrip strength)) (a), plasma predialysis urea (b), nPCR (c), glycine (1/glycine) (d) and taurine (log taurine) (e) in the BCAA‐glycine group (black squares and line) and in the glycine‐BCAA group (gray dots and line). The gray zone indicates the wash‐out period which occurred for each patient between month 4 and 5.
