# Supplementary material for: Glycine increases fat‐free mass in malnourished haemodialysis patients: a randomized double‐blind crossover trial
Source: J Cachexia Sarcopenia Muscle. 2021 Sep 14;12(6):1540–52. doi: 10.1002/jcsm.12780 (PMC8718019; doi:10.1002/jcsm.12780)
Supplement: Supplementary file 1 — Table S1. Study schedule detailing the frequency of the different assessments. Table S2. Differences of the outcomes between month 4 and 0, by supplementation. Table S3. Baseline plasma amino acid profile. Table S4. Multiple mixed linear regressions including period (first 4 months vs. following 4 months), supplementation (branched chain amino acids vs. glycine) and months as fixed effects, and subjects as random intercepts, to predict outcomes other than lean body mass index and fat‐free mass index (n = 27). Table S5. Multiple mixed linear regressions including period (first 4 months vs. following 4 months), supplementation (branched chain amino acids vs. glycine) and months as fixed effects, and subjects as random intercepts, to predict plasma amino acid concentrations (n = 26). Figure S1. Individual patient lines plots for body weight (A), lean body mass index (DXA) (B), fat mass index (DXA) (C), fat‐free mass index (BIA) (D) and fat‐mass index (BIA) (E). The gray line is used for the BCAA supplementation and the black line for the glycine supplementation. Plain lines correspond to the BCAA‐glycine group, while the dashed lines correspond to the Glycine‐BCAA group. Figure S2. Line plot showing the evolution of handgrip strength (squared root (handgrip strength)) (a), plasma predialysis urea (b), nPCR (c), glycine (1/glycine) (d) and taurine (log taurine) (e) in the BCAA‐glycine group (black squares and line) and in the glycine‐BCAA group (gray dots and line). The gray zone indicates the wash‐out period which occurred for each patient between month 4 and 5. [file JCSM-12-1540-s001.docx]

**SUPPLEMENTAL MATERIAL**

Supplemental table 1: Study schedule detailing the frequency of the different assessments

Supplemental table 2: Differences of the outcomes between month 4 and 0, by supplementation.

Supplemental table 3: Baseline plasma amino acid profile

Supplemental table 4: Multiple mixed linear regressions including period (first 4 months vs. following 4 months), supplementation (branched chain amino acids vs. glycine) and months as fixed effects, and subjects as random intercepts, to predict outcomes other than lean body mass index and fat-free mass index (n=27)

Supplemental table 5: Multiple mixed linear regressions including period (first 4 months vs. following 4 months), supplementation (branched chain amino acids vs. glycine) and months as fixed effects, and subjects as random intercepts, to predict plasma amino acid concentrations (n=26)

Supplemental figure 1: Individual patient lines plots for body weight (A), lean body mass index (DXA) (B), fat mass index (DXA) (C), fat-free mass index (BIA) (D) and fat-mass index (BIA) (E). The gray line is used for the BCAA supplementation and the black line for the glycine supplementation. Plain lines correspond to the BCAA-glycine group, while the dashed lines correspond to the Glycine-BCAA group.

Supplemental figure 2: Line plot showing the evolution of handgrip strength (squared root (handgrip strength)) (a), plasma predialysis urea (b), nPCR (c), glycine (1/glycine) (d) and taurine (log taurine) (e) in the BCAA-glycine group (black squares and line) and in the glycine-BCAA group (gray dots and line). The gray zone indicates the wash-out period which occurred for each patient between month 4 and 5. i

**Supplemental table 1: Study schedule detailing the frequency of the different assessments**

|  |  | BCAA or glycine | | | | | Wash-out | BCAA or glycine | | | | |
| --- | --- | --- | --- | --- | --- | --- | --- | --- | --- | --- | --- | --- |
| Assessments | Screening | Month 0 | Month 1 | Month 2 | Month 3 | Month 4 |  | Month 5 | Month 6 | Month 7 | Month 8 | Month 9 |
| Medical history |  | x | x | x | x | x |  | x | x | x | x | x |
| Medication | x | x | x | x | x | x |  | x | x | x | x | x |
| **Anthropometry** |  |  |  |  |  |  |  |  |  |  |  |  |
| Weight, height | x | x | x | x | x | x |  | x | x | x | x | x |
| Body composition (BIA) |  | x |  | x |  | x |  | x |  | x |  | x |
| Body composition (DXA) |  | x |  |  |  | x |  | x |  |  |  | x |
| **Laboratory parameters** |  |  |  |  |  |  |  |  |  |  |  |  |
| Hematology |  | x | x | x | x | x |  | x | x | x | x | x |
| Blood chemistry | x | x | x | x | x | x |  | x | x | x | x | x |
| **Intake and appetite** |  |  |  |  |  |  |  |  |  |  |  |  |
| 24-h food recall | x |  |  |  |  |  |  |  |  |  |  |  |
| 3-day food report |  | x |  | x |  | x |  | x |  | x |  | x |
| **Indirect calorimetry** |  | x |  |  |  | x |  | x |  |  |  | x |
| **Physical function** |  |  |  |  |  |  |  |  |  |  |  |  |
| Handgrip strength |  | x | x | x | x | x |  | x | x | x | x | x |
| Pedometry |  | x |  |  |  | x |  | x |  |  |  | x |
| **Quality of life** |  | x |  |  |  | x |  | x |  |  |  | x |

BCAA: branched-chain amino acids; BIA: bioelectrical impedance analysis; DXA: dual-energy x-ray absorptiometry

**Supplemental table 2:** Differences of the outcomes between month 4 and 0, by supplementation

|  | BCAA | | Glycine | |
| --- | --- | --- | --- | --- |
|  | Month 4 - Month 0 | | Month 4 - Month 0 | |
|  | mean | SD | mean | SD |
| **Anthropometry and body composition** |  |  |  |  |
| Body weight (kg) | 0.29 | 2.46 | 0.10 | 1.65 |
| Body mass index (kg/m^2^) | 0.10 | 0.87 | 0.03 | 0.63 |
| **Body composition** |  |  |  |  |
| DXA-Lean soft tissue (kg) | 0.22 | 2.42 | 0.55 | 1.71 |
| DXA-Fat mass (kg) | 0.13 | 1.73 | -0.32 | 1.55 |
| DXA-Bone mineral content (kg) | -0.14 | 0.67 | -0.01 | 0.06 |
| DXA-Lean body mass index (kg/m^2^) | 0.07 | 0.95 | 0.2 | 0.67 |
| DXA-Fat mass index (kg/m^2^) | 0.04 | 0.63 | -0.12 | 0.6 |
| BIA-Fat-free mass (kg) | -0.14 | 1.49 | 1.03 | 2.26 |
| BIA-Fat mass (kg) | 0.49 | 2.19 | -0.94 | 2.63 |
| BIA-Fat-free mass index (kg/m^2^) | -0.06 | 0.56 | 0.39 | 0.83 |
| BIA-Fat mass index (kg/m^2^) | 0.18 | -1.18 | -0.37 | 0.96 |
| **Blood parameters** |  |  |  |  |
| Hemoglobin (g/l) | 2.44 | 17.10 | 0.56 | 12.47 |
| Predialysis urea (mmol/l) | 1.84 | 4.68 | 2.39 | 5.97 |
| Creatinin (umol/l) | 14.78 | 145.84 | 27.89 | 94.56 |
| nPCR | 0.09 | 0.24 | 0.11 | 0.23 |
| Kt/Vurea | -0.05 | 0.56 | 0.03 | 0.35 |
| Bicarbonate (mmol/l) | -0.24 | 2.21 | 0.21 | 2.44 |
| Albumin (g/l) | 0.42 | 2.42 | 0.07 | 3.77 |
| Prealbumin (mg/l) | 18.67 | 42.76 | -19.04 | 57.09 |
| C-reactive protein (g/l) | -6.12 | 14.21 | 1.63 | 13.67 |
| Cholesterol (mmol/l) | 0.06 | 0.60 | -0.12 | 0.7 |
| Parathyroid hormone (pmol/l) | 6.73 | 35.34 | -1.15 | 233.94 |
| 25-OH vitamin D (nmol/l) | 3.38 | 19.22 | 2.20 | 25.85 |
| **Intake and appetite** |  |  |  |  |
| Kcalories (kcal/kg) | -12.67 | 289.78 | -187.95 | 3376.3 |
| Protein (g/kg) | -2.09 | 14.36 | -6.78 | 23.44 |
| Appetite rating (mm) | 0.48 | 1.67 | 0.26 | 1.45 |
| **Indirect calorimetry** |  |  |  |  |
| VCO2 (ml/min) | 11.11 | 31.05 | 2.11 | 29.85 |
| VO2 (ml/min) | 11.33 | 43.96 | 4.11 | 37.00 |
| Resting energy expenditure (kcal/d) | 79-96 | 287.4 | 26.00 | 248.65 |
| **Physical function** |  |  |  |  |
| Handgrip strength (kg) | 0.37 | 3.28 | 1.56 | 4.89 |
| Pedometry (steps/d) | -259.72 | 2006.36 | 337.29 | 1733.15 |
| **Quality of life** |  |  |  |  |
| General Health (0 to 100%) | -1.11 | 16.72 | 4.61 | 12.64 |
| Health change (0 to 100%) | -2.78 | 32.77 | 0.00 | 26.46 |

**Supplemental table 3: Baseline pre-dialysis plasma amino acid profile (µmol/l) in the fasting state**

|  | BCAA-glycine (n=15) | | Glycine-BCAA (n=11)^a^ | | p^b^ |
| --- | --- | --- | --- | --- | --- |
|  | median | (IQR) | median | (IQR) |  |
| Alanine | 317.0 | (279.0 - 473.0) | 352.0 | (266.0 - 385.0) | 0.725 |
| Arginine | 64.0 | (45.0 - 99.0) | 68.0 | (61.0 - 77.0) | 0.720 |
| Asparagine | 44.0 | (38.0 - 64.0) | 49.0 | (34.0 - 61.0) | 0.905 |
| Aspartate | 3.0 | (3.0 - 5.0) | 4.0 | (3.0 - 6.0) | 0.222 |
| Citrulline | 82.0 | (65.0 - 111.0) | 97.0 | (79.0 - 106.0) | 0.312 |
| Cystine | 78.0 | (54.0 - 89.0) | 91.0 | (88.0 - 104.0) | 0.006 |
| Glutamate | 58.0 | (48.0 - 89.0) | 83.0 | (57.0 - 98.0) | 0.007 |
| Glutamine | 530.0 | (456.0 - 566.0) | 497.0 | (442.0 - 549.0) | 0.342 |
| Glycine | 269.0 | (195.0 - 323.0) | 254.0 | (198.0 - 351.0) | 0.058 |
| Histidine | 64.0 | (51.0 - 78.0) | 67.0 | (61.0 - 75.0) | 0.658 |
| Isoleucine | 57.0 | (45.0 - 75.0) | 62.0 | (44.0 - 76.0) | 0.358 |
| Leucine | 99.0 | (77.0 - 114.0) | 99.0 | (92.0 - 124.0) | 0.404 |
| Lysine | 138.0 | (124.0 - 189.0) | 141.0 | (128.0 - 190.0) | 0.332 |
| Methionine | 21.0 | (18.0 - 27.0) | 23.0 | 17.0 - 27.0) | 0.521 |
| Ornithine | 68.0 | (58.0 - 84.0) | 66.0 | (59.0 - 80.0) | 0.855 |
| Phenylalanine | 62.0 | (55.0 - 75.0) | 69.0 | (52.0 - 78.0) | 0.156 |
| Proline | 281.0 | (222.0 - 333.0) | 269.0 | (218.0 - 368.0) | 0.778 |
| Serine | 71.0 | (49.0 - 74.0) | 63.0 | (55.0 - 91.0) | 0.013 |
| Taurine | 140.0 | (111.0 - 189.0) | 150.0 | (117.0 - 206.0) | 0.843 |
| Threonine | 97.0 | (82.0 - 138.0) | 113.0 | (98.0 - 139.0) | 0.431 |
| Tryptophane | 20.0 | (14.0 - 28.0) | 22.0 | (16.0 - 25.0) | 0.699 |
| Tyrosine | 37.0 | (33.0 - 57.0) | 40.0 | (34.0 - 49.0) | 0.447 |
| Valine | 202.0 | (145.0 - 241.0) | 196.0 | (165.0 - 223.0) | 0.350 |
| Total amino acids | 3066.0 | (2647.0 - 3211.0) | 3174.0 | (2676.0 - 3340.0) | 0.305 |

BCAA: branched-chain amino acids

^a^One blood sample missing

^b^Wilcoxon rank sum test. With the Benjamini-Hochberg method, significance was corrected to p<0.002, leaving no significant differences between groups.

**Supplemental table 4: Multiple mixed linear regressions including period, supplementation, months, age and sex as fixed effects, and subjects as random intercepts, to predict outcomes other than body weight, lean body mass index and fat-free mass index**

| Outcome | Normalized outcome | Coefficient | 95% CI | P |
| --- | --- | --- | --- | --- |
| **Body composition** |  |  |  |  |
| DXA-Fat mass index (kg/m^2^) |  | -0.05 | (-0.23, 0.13) | 0.603 |
| BIA-Fat mass index (kg/m2) |  | 0.06 | (-0.14, 0.26) | 0.578 |
| **Blood parameters** |  |  |  |  |
| Hemoglobin (g/l) |  | 1.04 | (-1.07, 3.15) | 0.336 |
| Pre-dialysis urea (mmol/l) |  | -0.53 | (-1.50, 0.45) | 0.289 |
| Creatinin (umol/l)* | sqrt (Creatinin) | -0.14 | (-0.49, 0.21) | 0.445 |
| nPCR |  | -0.04 | (-0.09, 0.01) | 0.106 |
| Kt/Vurea |  | 0.01 | (-0.07, 0.10) | 0.750 |
| Bicarbonate (mmol/l) |  | -0.46 | (-1.05, 0.13) | 0.128 |
| Albumin (g/l) |  | 0.10 | (-0.44 ,0.63) | 0.722 |
| Transthyretin (mg/l) |  | 14.10 | (5.38, 22.81) | 0.002 |
| C-reactive protein (g/l)* | log (C-reactive protein) | -0.26 | (-0.45, -0.08) | 0.005 |
| Cholesterol (mmol/l) |  | 0.05 | (-0.11, 0.21) | 0.554 |
| Parathyroid hormone (pmol/l)* | log (Parathyroid hormone) | 0.07 | (-0.10, 0.24) | 0.443 |
| 25-OH vitamin D (nmol/l) |  | 2.01 | (-3.76, 7.78) | 0.495 |
| **Intake and appetite** |  |  |  |  |
| Kilocalories (kcal/kg) |  | -0.86 | (-1.87, 0.15) | 0.095 |
| Protein (g/kg) |  | -0.04 | (-0.10, 0.01) | 0.109 |
| Appetite rating (mm) |  | 0.19 | (-0.05, 0.43) | 0.114 |
| **Indirect calorimetry** |  |  |  |  |
| VCO_2_ (ml/min) |  | 1.23 | (-9.01, 11.47) | 0.814 |
| VO_2_ (ml/min) |  | 2.19 | (-6.26, 10.65) | 0.611 |
| Respiratory quotient |  | 0.02 | (-0.02, 0.06) | 0.483 |
| Resting energy expenditure (kcal/d) |  | 10.07 | (-58.70, 78.83) | 0.774 |
| **Physical function** |  |  |  |  |
| Handgrip strength (kg)* | sqrt (Handgrip strength) | 0.06 | (0.00, 0.12) | 0.050 |
| Pedometry (steps/day)* | sqrt (Pedometry) | 1.79 | (-1.94, 5.53) | 0.346 |
| **Quality of life** |  |  |  |  |
| General Health (0 to 100%) |  | -0.53 | (-4.53, 3.47) | 0.795 |
| Health change (0 to 100%) |  | -5.24 | (-13.00, 2.53) | 0.186 |

95%CI: 95% confidence interval, sqrt: squared root

The coefficient, 95% CI and p value correspond to the impact of the branched chain amino acids vs. the glycine (reference). With the Benjamini-Hochberg method including analysis of these outcomes and those presented on table 3, significance for supplementation was corrected to p<0.005.

* Parameters with non-Gaussian distribution, which required normalization for use in muliple mixed linear regressions

**Supplemental table 5: Multiple mixed linear regressions including period, supplementation, months, age and sex as fixed effects, and subjects as random intercepts, to predict plasma amino acid concentrations (n=26)**

|  | |  | |  | |  | |  | |
| --- | --- | --- | --- | --- | --- | --- | --- | --- | --- |
| Outcome | Normalized Outcome | | Coefficient | | 95% CI | | p | |  |
| Alanine | Log (Alanine) | | -0.01 | | (-0.08, 0.08) | | 0.962 | |  |
| Arginine | Log (Arginine) | | 0.05 | | (-0.02, 0.12) | | 0.128 | |  |
| Asparagine | Log (Asparagine) | | 0.03 | | (-0.03, 0.09) | | 0.285 | |  |
| Aspartate | Log (Aspartate) | | -0.16 | | (-0.29,-0.02) | | 0.028 | |  |
| Citrulline | 1/sqrt (Citrulline) | | -0.01 | | (-0.01, 0.01) | | 0.577 | |  |
| Cystine | sqrt (Cystine) | | -0.11 | | (-0.41, 0.19) | | 0.482 | |  |
| Glutamate | sqrt (Glutamate) | | -0.13 | | (-0.42, 0.17) | | 0.409 | |  |
| Glutamine |  | | 2.38 | | (-14.50, 19.27) | | 0.782 | |  |
| Glycine | 1/Glycine | | 0.01 | | (-0.01, 0.01) | | 0.072 | |  |
| Histidine |  | | 1.72 | | (-1.67, 5.11) | | 0.320 | |  |
| Isoleucine | 1/sqrt (Isoleucine) | | -0.01 | | (-0.01, 0.01) | | 0.134 | |  |
| Leucine | 1/sqrt (Leucine) | | -0.01 | | (-0.12, 0.01) | | 0.057 | |  |
| Lysine | Log (Lysine) | | 0.02 | | (-0.05, 0.09) | | 0.542 | |  |
| Methionine | 1/sqrt (Methionine) | | -0.01 | | (-0.01, 0.01) | | 0.378 | |  |
| Ornithine | Log (Ornithine) | | 0.06 | | (-0.01, 0.12) | | 0.067 | |  |
| Phenylalanine | 1/Phenylalanine | | -0.01 | | (-0.01, 0.01) | | 0.132 | |  |
| Proline | Log (Proline) | | -0.06 | | (-0.14, 0.02) | | 0.163 | |  |
| Serine | 1/sqrt (Serine) | | 0.01 | | (-0.01, 0.01) | | 0.052 | |  |
| Taurine | Log (Taurine) | | -0.01 | | (-0.08, 0.07) | | 0.925 | |  |
| Threonine | sqrt (Threonine | | 0.14 | | (-0.28, 0.57) | | 0.499 | |  |
| Tryptophane | Log (Tryptophane) | | 0.06 | | (-0.02, 0.14) | | 0.194 | |  |
| Tyrosine | 1/sqrt (Tyrosine) | | -0.01 | | (-0.01, -0.01) | | 0.273 | |  |
| Valine | 1/sqrt (Valine) | | -0.01 | | (-0.01, -0.01) | | 0.041 | |  |
| Total amino acids | 1/total amino acids | | 0.01 | | (-0.01, 0.01) | | 0.568 | |  |

95%CI: 95% confidence interval.

All parameters besides plasma glutamine and histidine had a non-Gaussian distribution, and required normalization for use in multiple mixed linear regressions.

The coefficient, 95% CI and p value correspond to the impact of the branched chain amino acids vs. the glycine (reference). With the Benjamini-Hochberg method, significance for supplementation was corrected to p<0.002.

**Supplemental figure 1**

**A.**

**B.**

**C.**

**D.**

**E.**

**Supplemental figure 2**

a.

Glycine

BCAA

Glycine

Glycine

Glycine

BCAA

BCAA

BCAA

Glycine

Glycine

BCAA

BCAA

Period 1

Period 2

Period 1

Period 2

Period 1

Period 2

b.

c.
